# Two new species of cricket genus *Anaxiphomorpha* Gorochov, 1987 (Orthoptera, Trigonidiidae, Trigonidiinae) in China

**DOI:** 10.3897/zookeys.1073.75598

**Published:** 2021-11-29

**Authors:** Zhi Xin He, Li Bin Ma

**Affiliations:** 1 Shaanxi Normal University, 710119 Xi’an, China Shaanxi Normal University Xi’an China

**Keywords:** Southern China, swordtail crickets, taxonomy, Trigonidiini, tropics

## Abstract

Two new species, *Anaxiphomorphanonggangensis***sp. nov.** and *Anaxiphomorphamanereserratus***sp. nov.**, are reported from Guangxi Province, China. Descriptions and illustrations for the new species and a key to all known species of *Anaxiphomorpha* are provided.

## Introduction

The genus *Anaxiphomorpha* was established with *Anaxiphomorphabrachyapodemalis* Gorochov, 1987 as the type species. *Anaxiphomorpha* is recognized by a smaller body size, yellow coloration and special genitalia structure (epiphallus and ectoparamere possess multiple lobes or branches).

**Figure 1.**
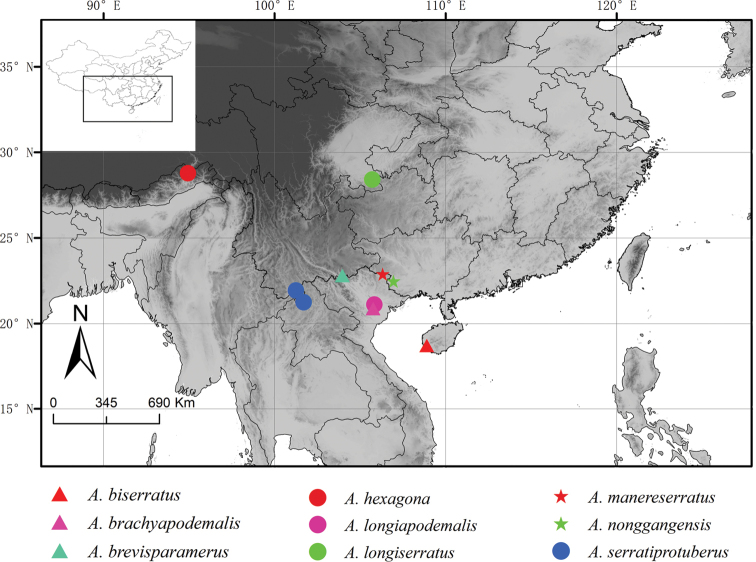
Known distribution of *Anaxiphomorpha* species, including the new species *A.nonggangensis* sp. nov. and *A.manereserratus* sp. nov..

To date, seven species have been reported worldwide (Cigliano et al. 2021). Apart from two species, *Anaxiphomorphabrachyapodemalis* Gorochov, 1987 and *Anaxiphomorphalongiapodemalis* Gorochov, 1987 reported from Vietnam, the other five, *Anaxiphomorphabiserratus* Liu & Shi, 2015, *Anaxiphomorphabrevisparamerus* Liu & Shi, 2015, *Anaxiphomorphalongiserratus* Liu & Shi, 2015, *Anaxiphomorphaserratiprotuberus* Liu & Shi, 2015 and *Anaxiphomorphahexagona* Ma, 2018 have been recorded from China. After comparing our new materials with the species of this genus, we concluded that two Chinese species are new for science. The distribution of *Anaxiphomorpha* species worldwide including the new species is also presented (Fig. 1).

## Materials and methods

All specimens were collected at night with a sweep net. Specimens were preserved in ethanol during field work, and pinned and dry-preserved in the laboratory. Male genitalia were dissected from softened specimens. Photomicrographs of genitalia were collected using ToupCam Digital camera and bundled software (ToupTek, Hangzhou, China). Photographs of specimens were obtained using a VHX–6000 digital microscope (Keyence, Osaka, Japan).

### Measurements

All specimens were measured using a ToupCam Digital camera and bundled software (ToupTek, Hangzhou, China). All the measurements are in millimeters (mm).

### Abbreviations

**BL** body length (from head to apical hindwing);

**PL** pronotal length;

**TL** tegmen length;

**HFL** hind femur length;

**OL** ovipositor length.

The specimens are deposited at the Museum of Flora and Fauna of Shaanxi Normal University, Xi’an, China (SNNU).

## Taxonomy

### Genus Anaxiphomorpha

Taxon classificationAnimaliaOrthopteraGryllidae

Gorochov, 1987

8374FB5F-8FE4-5CDD-BF28-6E59F3CBD83D

#### Type species.

*Anaxiphomorphabrachyapodemalis* Gorochov, 1987

### Key to known species of *Anaxiphomorpha*

**Table:** 

1	Dorsally viewed, epiphallic lateral lobes apically acute	**2**
–	Dorsally viewed, epiphallic lateral lobes somewhat blunt of apex	**3**
2	Epiphallic transverse bridge and ectoparamere short	***A.brachyapodemalis***
–	Epiphallic transverse bridge and ectoparamere long	***A.longiapodemalis***
3	Epiphallic lateral lobes bifurcated as six significant branches	***A.hexagona***
–	Not as above and laterally viewed as following	**4**
4	Apex of epiphallic lateral lobes almost straight	***A.brevisparamerus***
–	Apex of epiphallic lateral lobes curved	**5**
5	Apex of epiphallic lateral lobes upward curved	**6**
–	Apex of epiphallic lateral lobes downward curved	**8**
6	Epiphallic lateral lobes boot-like	***A.nonggangensis* sp. nov.**
–	Epiphallic lateral lobes rod-like	**7**
7	Epiphallic lateral lobes proximally bearing a protuberance	***A.manereserratus* sp. nov.**
–	Not as above	***A.longiserratus***
8	Epiphallic lateral lobes long, medially raised and apically acute	***A.serratiprotuberus***
–	Epiphallic lateral lobes short and apically blunt	***A.biserratus***

### Anaxiphomorpha nonggangensis

Taxon classificationAnimaliaOrthopteraGryllidae

He & Ma sp. nov.

2060E671-CD52-5647-B649-5D92190217A1

http://zoobank.org/960B75C1-468B-4D24-BADE-06F8D7F93D2E

**Figure 2.**
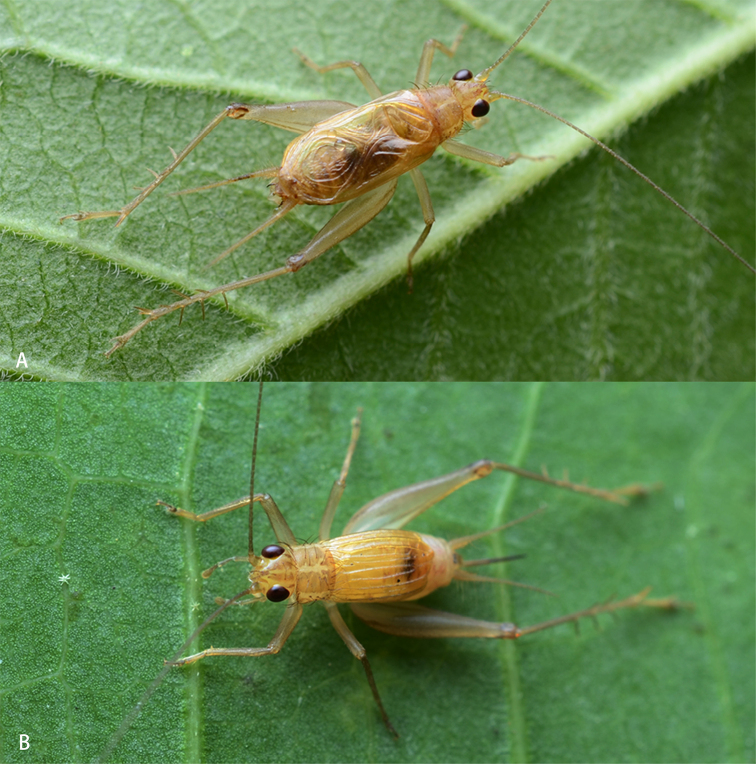
Habitus (alive) of *A.nonggangensis* sp. nov. on leaf **A** male **B** female (photography: Zhang, Tao).

**Figure 3.**
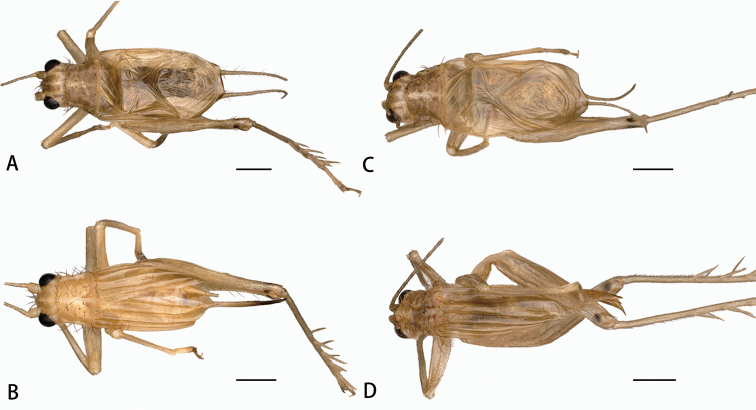
Habitus photographs. **A, B***A.nonggangensis* sp. nov. **C, D***A.manereserratus* sp. nov. **A, C** Male **B, D** Female. Scale bar: 1 mm.

**Figure 4.**
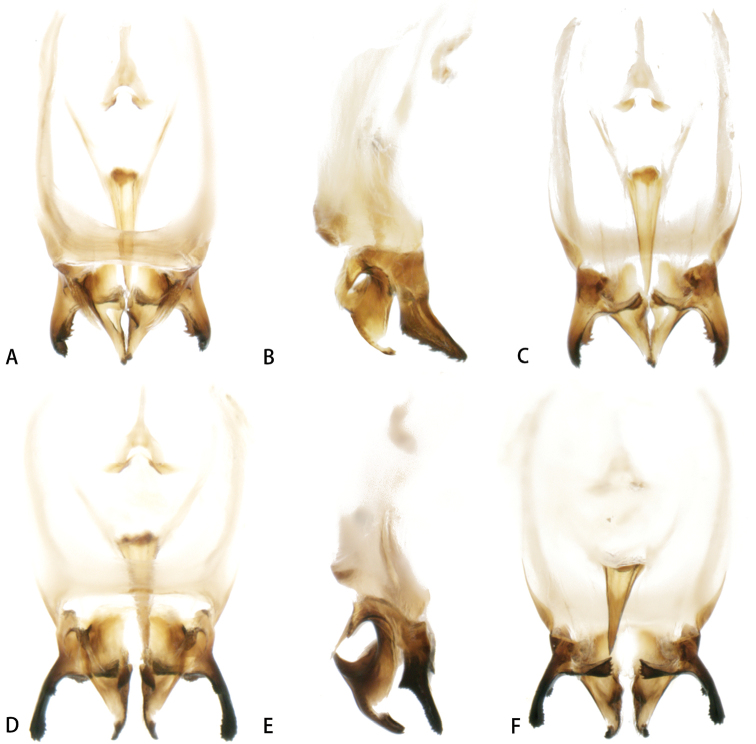
Male genitalia **A–C***A.nonggangensis* sp. nov. **D–F***A.manereserratus* sp. nov. **A, D** dorsal views **B, E** lateral views **C, F** ventral views.

Figs 1 , 2 , 3A, B , 4A–C

#### Material examined.

***Holotype*.** China: Male, Guangxi, Longzhou, Nonggang National Natural Reserve, 5.V.2019, 22.46°N, 106.96°E, Libin Ma & Tao Zhang leg. ***Paratypes*.** 7 males and 4 females, same information as holotype (SNNU).

#### Diagnosis.

**Male (Figs 2A, 3A).** Body size small. Head small and pubescent, slightly wider than anterior margin of pronotum; frontal rostrum as wide as antennal scape; eyes large and protruding forwards; apical three joints of maxillary palpi distinctly elongate, 5^th^ joint truncated apically. Pronotum transverse, strongly widened posteriorly and distinctly longer than width of anterior one. Tegmina extending slightly over apex of abdomen, armed with one oblique vein; mirror slightly elongate; hindwings absent. Fore tibia armed with a large long-oval external tympanum, and without internal tympanum. Hind tibia bearing three dorsal spurs on two sides respectively, and bearing two apical spurs inside and three outside.

***Genitalia*** (Fig. 4A–C). Lateral lobes of epiphallus slightly longer than median lobes, and possessing several teeth at outer margin, not narrowed apically in dorsal view, gradually narrowed apically in lateral view; median lobes shaped as boot and abruptly narrowed apically in lateral view. Etcoparameres transversely and truncated apically.

**Female (Figs 2B, 3B).** Body slightly smaller than male. Tegmen rather convex, armed with five regular veins on dorsal field. Ovipositor blade-shaped.

#### Coloration.

Body colored yellow. Dorsal area of head ornamented with four brown longitudinal stripes in ventral view, anterior half of abdomen colored dark brown in male or small part of middle abdomen colored dark in female. Apex of each hind femur bearing a small dark spot on two sides respectively. Ovipositor ventrally colored brown to dark brown and remainder yellowish.

#### Measurements.

**Male.**BL 5.17–6.42, PL 0.98–1.19, TL 3.73–4.54, HFL 3.27–3.96. **Female.**BL 4.50–5.63, PL 0.99–1.17, TL 3.87–4.42, OL 2.22–2.67.

#### Etymology.

The name refers its type locality, Nonggang National Natural Reserve.

#### Distribution.

China (Guangxi) (Fig. 1).

#### Remarks.

This species is very similar to *A.brevisparamerus* in the ectoparameres of the male genitalia, but different in the epiphallic lateral lobes of this new species, which are shaped as boot with an acute apex.

### Anaxiphomorpha manereserratus

Taxon classificationAnimaliaOrthopteraGryllidae

He & Ma sp. nov.

57816F8B-B63B-5C0B-9C72-C3E8F5921B3A

http://zoobank.org/9B5380BF-9334-4480-B796-4FE6DF673DB1

**Figure 5.**
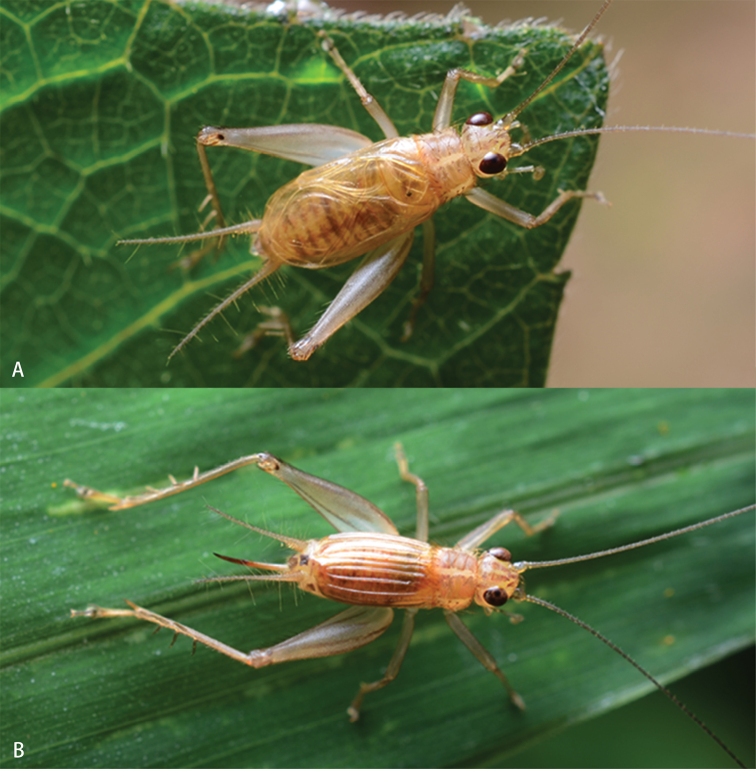
Habitus (alive) of *A.manereserratus* sp. nov. on leaf **A** male **B** female (photography: Zhang, Tao).

Figs 1 , 3C–D , 4D–F , 5

#### Material examined.

***Holotype*.** China: Male, Guangxi, Jingxi, Longbang, 22.87°N, 106.32°E, 1.V.2019, Libin Ma & Tao Zhang leg. ***Paratypes*.** 10 males and 2 females, same information as holotype (SNNU).

#### Description.

**Male (Figs 3C, 5A).** Body size small. Head small and pubescent, slightly wider than anterior margin of pronotum; frontal rostrum 1.2 times wider than antennal scape; eyes large and protruding forwards; apical three joints of maxillary palpi distinctly elongate, 5^th^ joint truncated apically. Pronotum transverse, strongly widened posteriorly, and slightly wider than the anterior. Tegmina extending slightly over apex of abdomen, armed with one oblique vein; mirror slightly elongate; hindwings absent. Fore tibia armed with a large long-oval external tympanum; internal tympanum absent. Hind tibia bearing three dorsal spurs on two sides respectively, and bearing two apical spurs inside and three outside.

***Genitalia*** (Fig. 4D–F). Lateral lobes of epiphallus slightly longer than median lobes in lateral view and possessing horned protuberances at inner margin proximally and outer margin medially, bearing several teeth at inner and outer margin and not narrowed apically in lateral view. Ectoparameres short and serrated apically.

**Female (Figs 3D, 5B).** Body slightly smaller than male. Tegmen rather convex, armed with five regular veins on dorsal field. Ovipositor blade-shaped.

#### Coloration.

Body colored yellow. Dorsal area of head ornamented with four brown longitudinal stripes. Apex of each hind femur bearing a small dark spot in two sides respectively. Ovipositor ventrally colored brown to dark brown and remainder yellowish.

#### Measurements.

**Male.**BL 4.92–6.05, PL 0.94–1.15, TL 3.42–4.09, HFL 3.56–4.17. **Female.**BL 4.65–4.94, PL 0.87–0.90, TL 3.21–3.33, OL 1.892.03.

#### Etymology.

The name refers to the epiphallic median lobes almost as long as the lateral lobes.

#### Distribution.

China (Guangxi) (Fig. 1).

#### Remarks.

This species is very similar to *A.serratiprotuberus* and *A.longiserratus* in dorsal and ventral views of the male genitalia, but different in lateral view (epiphallic lateral lobes of the new species possessing horned protuberances at the inner margin proximally and the outer margin medially, and the ectoparameres of this new species are acute apically).

## Supplementary Material

XML Treatment for Anaxiphomorpha

XML Treatment for Anaxiphomorpha nonggangensis

XML Treatment for Anaxiphomorpha manereserratus
